# Comparative safety analysis of immunotherapy combined with chemotherapy versus monotherapy in solid tumors: a meta-analysis of randomized clinical trials

**DOI:** 10.18632/oncotarget.26908

**Published:** 2019-05-14

**Authors:** Alberto Carretero-González, David Lora, Ismael Ghanem, Irene Otero, Flora López, Daniel Castellano, Guillermo de Velasco

**Affiliations:** ^1^ Medical Oncology Department, University Hospital 12 de Octubre, Madrid, Spain; ^2^ Clinical Research Unit (imas12-CIBERESP), University Hospital 12 de Octubre, Madrid, Spain; ^3^ Medical Oncology Department, University Hospital La Paz, Madrid, Spain

**Keywords:** immunotherapy, chemotherapy, combination treatment, safety profile, adverse events

## Abstract

**Background:** Combination treatment (chemotherapy plus immune checkpoint blockade [ICB]) has shown promising activity in terms of efficacy, but it has been suggested that its toxicity profile is less favorable compared to monotherapy.

**Methods:** We conducted a meta-analysis of published randomized clinical trials comparing combination treatment to monotherapy (chemotherapy or ICB) in patients with metastatic solid tumors. Differences in rates of safety issues (all-grade adverse events, grade 3/4 adverse events, treatment-related deaths, treatment discontinuations) between groups were estimated. Subgroup analyses for the control group (chemotherapy or ICB as monotherapy) and immune checkpoint inhibitor (anti-CTLA-4 or anti-PD-1/PD-L1 antibodies) were performed.

**Results:** A total of 4379 patients (ten studies) were included (monotherapy: 2026 patients; combination treatment: 2353 patients). Combination treatment presented more grade 3/4 adverse events (RR 1.32, 95% CI 1.12–1.55) and discontinuations (RR 2.31, 95% CI 1.28–4.16). There were no differences in the mortality rate between groups. Subgroup analyses showed a potentially more toxic profile with anti-CTLA-4 agents.

**Conclusions:** Combination treatment is associated with an increase in grade 3/4 adverse events and treatment discontinuations compared to monotherapy, but not increased mortality. The toxicity profile of combination therapy should be considered with regard to the overlapping safety profiles.

## INTRODUCTION

Immunotherapy has become one of the best strategies to treat several tumors in recent years. Immune checkpoint blockade (ICB) through anti-Programmed Cell Death-1 (anti-PD-1), anti-Programmed Cell Death-Ligand 1 (anti-PD-L1) and anti-Cytotoxic T Lymphocyte-associated Antigen-4 (anti-CTLA-4) monoclonal antibodies (mAbs) represents a way of modulating the immune system, with remarkable outcomes.

Unfortunately, a significant number of patients do not benefit from ICB, and up to 40% of patients treated with ICB obtain a status of progressive disease (PD) as best response with these anti-PD-1/PD-L1/CTLA-4 mAbs [1–6].

Different molecular mechanisms of resistance to immunotherapy are being elucidated, from which some actionable strategies to prevent or treat them could be derived. Most contemporary approaches focus on combination strategies in an effort to overcome resistance to monotherapy [7]. Combined therapy with blocking antibodies against key immune checkpoints, mainly CTLA-4 and PD-1 (but also anti-VISTA, anti-LAG-3 or anti-TIM-3 mAbs), constitutes an example of potential enhanced efficacy. For example, the combination nivolumab plus ipilimumab results in higher response rates compared to nivolumab or ipilimumab as monotherapy, and improved overall survival in patients with metastatic melanoma [8, 9]. Traditional chemotherapy (CTx) is also being combined with ICB strategies. The safety profile is probably the most common concern in treating patients with these combinations. ICB monotherapy has shown a better toxicity profile compared to CTx, with lower all-grade and grade 3/4 adverse events. Nevertheless, it is associated with a small but significant increase in the risk of selected all-grade immune-related adverse events and high-grade gastrointestinal and liver toxicities [10]. Overall, immune checkpoint inhibitors present a manageable safety profile, obtaining good outcomes by the implementation of dose interruptions and use of steroids when required. Combination treatments appear to have a less favorable safety profile compared with the monotherapy strategy. Combination treatment with CTx plus ICB could be associated with more high-grade adverse events and higher treatment discontinuation rates, similar to the pattern observed with ipilimumab plus nivolumab [8].

While combination therapies have the potential to achieve significant improvements in efficacy, their effectiveness has been derived from empirically chosen combinations, which can sometimes lead to higher toxicity as well as treatment discontinuations that may result in worse outcomes.

The safety profile of CTx plus ICB is not completely understood compared to monotherapy. We therefore performed a meta-analysis of published randomized clinical trials (RCT) in order to analyze the main safety endpoints (treatment-related adverse events, deaths and discontinuations) obtained with the combination of CTx plus ICB (anti-PD-1/PD-L1/CTLA-4 mAbs) compared to each treatment as monotherapy (CTx or ICB).

## PATIENTS AND METHODS

### Literature search and inclusion criteria

We identified all RCTs that compared combined treatments (CTx plus ICB, which comprises the concurrent use of CTx along with ICB) with CTx agents or currently approved immune checkpoint inhibitors (anti-PD-1, anti-PD-L1 or anti-CTLA-4 mAbs) as monotherapy. The immune checkpoint inhibitors included in this study were: nivolumab (Opdivo^®^), pembrolizumab (Keytruda^®^), atezolizumab (Tecentric^®^), durvalumab (Imfinzi^®^), avelumab (Bavencio^®^), ipilimumab (Yervoy^®^) and tremelimumab. An independent search of published studies from January 1, 2010 to July 1, 2018 in MEDLINE and EMBASE was performed. The following search terms were used: (“nivolumab” OR “pembrolizumab” OR “atezolizumab” OR “durvalumab” OR “avelumab” OR “ipilimumab” OR “tremelimumab”) AND (“trial” OR “randomized”). A parallel search entering the names of the agents and filtering the results by clinical trial type was conducted. The review was restricted to RCTs in human subjects published in English. We reviewed each publication, and only the most recent or complete report of RCTs was included when duplicate publications were identified. On July 1, 2018, the online updated manufacturers’ package inserts for nivolumab, pembrolizumab, atezolizumab, durvalumab, avelumab, ipilimumab and tremelimumab were also reviewed to identify relevant information not previously reported in published clinical trials. Trials involving combinations of ICB with agents other than CTx (mainly between two different immune checkpoint inhibitors) were excluded in order to reduce the potential heterogeneity among the results. Selected safety endpoints included: rates of adverse events (AEs; both all-grade and grade 3/4 adverse events), treatment-related deaths (deaths), and treatment discontinuations (discontinuations). Trials that met the following criteria were included in the meta-analysis: randomized phase I, II and III trials, prospective clinical trials in patients with cancer, and trials with at least one of the previous safety endpoints mentioned above available. Only studies with solid tumors were included. Two reviewers (A. C-G. and G.d.V.) independently evaluated studies for eligibility.

### Data extraction and clinical endpoints

Data were extracted as already outlined, after a preliminary screen by two investigators (A. C-G. and G. d. V.) according to Quality of Reporting of Meta-Analyses (QUORUM) guidelines. Variables collected and included were: first author’s surname, year of publication, National Clinical Trials (NCT) registry number, study phase, type of underlying malignancy, number of previous treatments received, selection of population by PD-L1 expression on tumor cells (yes/no), number of enrolled subjects, number of patients available for safety analysis, criteria used for grading adverse events, blinding (yes/no), treatment arms, number of patients per treatment arm, name of the immune checkpoint inhibitor (nivolumab, pembrolizumab, atezolizumab, durvalumab, avelumab, ipilimumab, tremelimumab), and median age. The safety endpoints selected for the analysis (% all-grade AEs, grade 3/4 AEs, deaths and discontinuations) were also obtained per treatment group to compare outcomes with the combination versus monotherapy.

### Statistical analysis

All statistical analyses were performed using meta package [11, 12]. For binary outcomes, all-grade AEs/grade 3/4 AEs/deaths/discontinuations risk ratios with confidence intervals (CIs) were used as the measure of safety of the CTx plus ICB arm versus the monotherapy (either CTx or ICB) arm (control arm). Statistical heterogeneity among trials included in the meta- analysis was assessed using Higgins´s I^2^ statistics, which estimates the percentage of total variation across studies due to heterogeneity rather than chance [13], and Cochran´s Q test with under the null hypothesis of no heterogeneity. We pooled studies using random and fixed-effects models depending on the heterogeneity of the studies included. When substantial heterogeneity was not observed, the summary estimate calculated on the basis of the fixed-effects model was reported using the Mantel-Haenszel method; otherwise, the random effects model was reported using the DerSimonian and Laird method that considers both within-study and between-study variations [14]. Subgroup analyses were conducted by control group (CTx or ICB as monotherapy) and by class of immune checkpoint inhibitor evaluated (in the monotherapy and/or combination treatment arms: anti-CTLA-4 mAb trials or anti-PD-1/PD-L1 mAb trials). As in the overall analysis, the purpose of these subgroup analyses was to compare monotherapy versus combination treatment in different subsets of populations. In addition, publication bias was evaluated using funnel plots (i.e. plots of study results against precision).

## RESULTS

### Study selection

**Figure 1:**
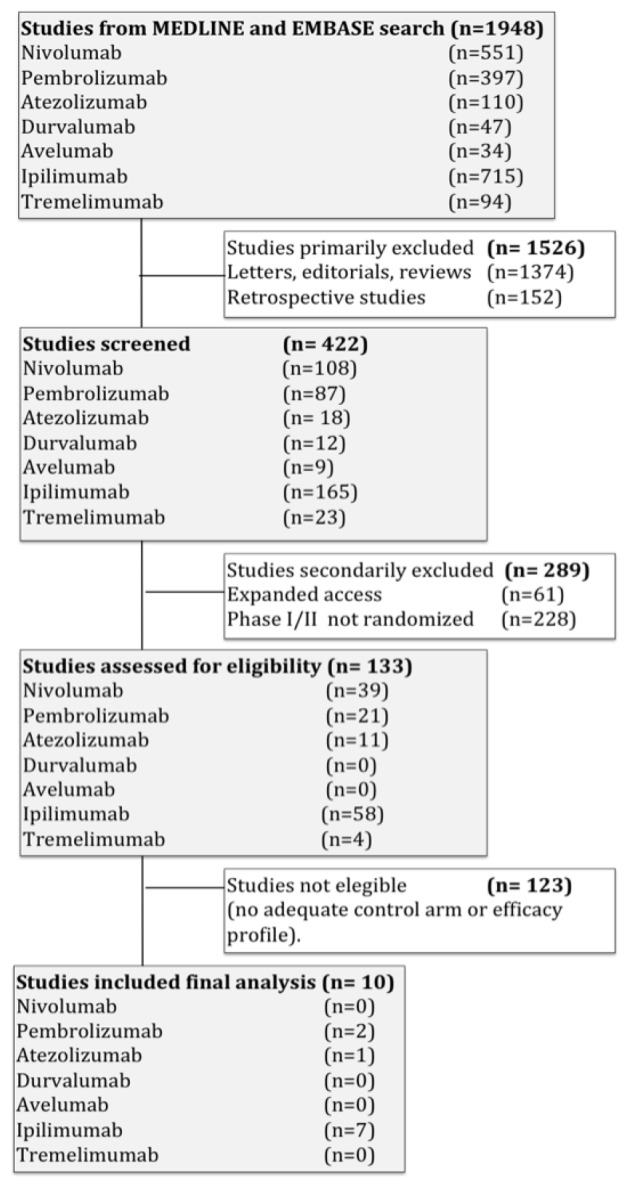
Flow diagram of the systematic review.

Studies selected are shown in the flow chart (Figure 1). A total of 1948 studies were reviewed through our screening process for RCTs. Exclusions were: (i) letters, editorials, reviews and retrospective studies (1526 studies); (ii) expanded-access studies with no control arm and early phase I/II or non-RCTs (289 studies); and (iii) studies with only efficacy results or no adequate control arm (123 studies). Ten trials met the criteria for inclusion in the meta-analysis (randomized phase I/II/III trials with CTx plus ICB compared to CTx/ICB in monotherapy as the control arm). As explained above, studies were carried out in patients with different metastatic solid tumors.

The baseline characteristics of each trial are presented in Table 1 [15–24]. Five trials were performed in patients with non-small cell lung cancer, two in patients with small cell lung cancer and three in melanoma. All were performed in the first-line setting. All studies had two treatment arms except three, which had three arms [16, 17, 22]. The IMpower150 study has three treatment arms, but results for only two of them have been published to date (results for atezolizumab combined with paclitaxel and carboplatin are pending) [24]. In two studies, ICB monotherapy was the control arm (ipilimumab) [20, 22]; the remaining studies used CTx as monotherapy (usually combined cytotoxic agents). Seven studies included anti-CTLA-4 mAbs (ipilimumab) and three studies included anti-PD-1/PD-L1 mAbs (pembrolizumab and atezolizumab). None of the studies selected populations according to PD-L1 expression on tumor cells or immune cells. Some of the studies used PD-L1 expression criteria as a stratification factor but no independent results have been published. A total of 4598 patients were available for the meta-analysis, 4379 of whom had safety data available: 2026 patients were assigned to monotherapy (1967 to combined chemotherapy regimens, 59 to ipilimumab), and 2353 were assigned to CTx plus ICB arms (1496 including ipilimumab, 464 including pembrolizumab, and 393 including atezolizumab). Four of these studies did not specify a relationship between at least one selected safety endpoint and the administered treatment (no treatment-related data available) [16, 21–23]. Safety was a secondary endpoint in all of the studies. Common Terminology Criteria for Adverse Events (CTCAE) was the criteria used to assess and grade adverse events (mainly versions 3.0 and 4.0). All RCTs were sponsored by pharmaceutical companies.

**Table 1:** List of clinical trials included in the meta-analysis

	Reference	Phase	Histology	Masking	No. patients (safety data)	Treatment arms
1	Reck M, *et al.* (2016)	3	Small cell lung cancer (SCLC)	Double-blind	954	Platinum + Etoposide + Placebo
						Platinum + Etoposide + Ipilimumab
2	Reck M, *et al.* (2012)	2	SCLC	Double-blind	128	Paclitaxel + Carboplatin + Placebo
						(Paclitaxel + Carboplatin + Placebo) followed by (Paclitaxel + Carboplatin + Ipilimumab)
						(Paclitaxel + Carboplatin + Ipilimumab) followed by (Paclitaxel + Carboplatin + Placebo)
3	Lynch TJ, *et al.* (2012)	2	Non-small cell lung cancer	Double-blind	203	Paclitaxel + Carboplatin + Placebo
						(Paclitaxel + Carboplatin + Placebo) followed by (Paclitaxel + Carboplatin + Ipilimumab)
						(Paclitaxel + Carboplatin + Ipilimumab) followed by (Paclitaxel + Carboplatin + Placebo)
4	Govindan R, *et al.* (2017)	3	NSCLC (Squamous-Sq-)	Double-blind	948	Paclitaxel + Carboplatin + Placebo
						(Paclitaxel + Carboplatin + Placebo) followed by (Paclitaxel + Carboplatin + Ipilimumab)
5	Langer CJ, *et al.* (2016)	2	NSCLC (Non-Sq)	Open-label	121	Carboplatin + Pemetrexed
						Carboplatin + Pemetrexed + Pembrolizumab
6	Hersh EM, *et al*. (2011)	2	Melanoma	Open-label	74	Ipilimumab
						Ipilimumab + Dacarbazine
7	Robert C, *et al.* (2011)	3	Melanoma	Double-blind	498	Placebo + Dacarbazine
						Ipilimumab + Dacarbazine
8	Weber J, *et al.* (2013)	1	Melanoma	Open-label	59	Ipilimumab
						Ipilimumab + Dacarbazine
						Ipilimumab + Paclitaxel + Carboplatin
9	Gandhi L, *et al.* (2018)	3	NSCLC (Non-Sq)	Double-blind	607	Platinum + Pemetrexed + Placebo
						Platinum + Pemetrexed + Pembrolizumab
10	Socinski MA, *et al.* (2018)	3	NSCLC (Non-Sq)	Open-label	787	Bevacizumab + Paclitaxel + Carboplatin
						Atezolizumab + Bevacizumab + Paclitaxel + Carboplatin
						Atezolizumab + Paclitaxel + Carboplatin (results not reported)

### Incidence and relative risk of all-grade AEs and grade 3/4 AEs

**Figure 2:**
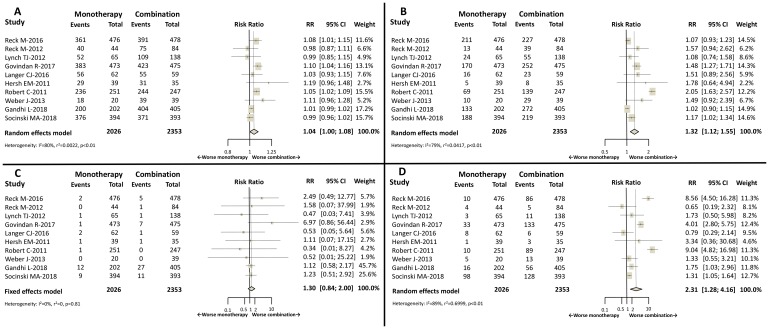
Forest plot diagrams: Relative risk (RR) with 95% confidence interval (CI) of safety endpoints between combination treatment and monotherapy. (**A**) All-grade AEs. (**B**) Grade 3/4 AEs. (**C**) Deaths. (**D**) Discontinuations.

In patients receiving CTx plus ICB, all-grade AEs were confirmed in 2142/2353 patients (91.03%) compared to 1751/2026 (86.43%) in those patients on monotherapy [Relative risk (RR) 1.04; 95% CI 1.00-1.08, *p* = 0.048 (Figure 2A)].

Grade 3/4 AEs were reported in 1263/2353 (53.68%) patients receiving CTx plus ICB, compared to 839/2026 (41.41%) in patients treated with monotherapy. An increased risk of grade 3/4 AEs was shown in patients treated with CTx plus ICB: RR 1.32; 95% CI 1.12–1.55, *p* = 0.0008 (Figure 2B). Two studies included did not specify whether the AEs were related or not to the study treatments [21, 23].

### Incidence and relative risk of deaths

Deaths were notified in 54/2353 (2.30%) patients treated with CTx plus ICB, while this event was observed in 29/2026 (1.43%) of patients receiving treatment as monotherapy. No differences were found between groups: RR 1.30; 95% CI 0.84-2.00, *p* = 0.24 (Figure 2C). One study did not specify the relationship between deaths and study treatments [23].

### Incidence and relative risk of discontinuations

Treatment discontinuations were reported in 530/2353 (22.52%) patients who received CTx plus ICB, and in 188/2026 (9.28%) patients managed with monotherapy. CTx plus ICB was associated with higher rate of discontinuations compared to monotherapy: RR 2.31; 95% CI 1.28-4.16, *p* = 0.006 (Figure 2D).

### Subgroup analyses (Table 2)

According to the monotherapy control arm (CTx or ICB), results were similar to the overall analysis, with no significant differences observed between these groups in any of the safety endpoints compared with CTx plus ICB.

**Table 2:** Subgroup analysis according to monotherapy control arm (chemotherapy or immunotherapy) and class of immune checkpoint inhibitor (anti-CTLA-4 mAb or anti-PD-1/PD-L1 mAb)

Subgroup analysis by control arm in monotherapy	Chemotherapy in monotherapy	Immunotherapy in monotherapy	*p*-value for difference
All grade-AEs	RR 1.03; 95% CI 0.99–1.07	RR 1.13; 95% CI 1.00–1.28	0.15
Grade 3/4 AEs	RR 1.30; 95% CI 1.09–1.54	RR 1.54; 95% CI 1.00–2.36	0.48
Deaths	RR 1.32; 95% CI 0.85–2.05	RR 0.87; 95% CI 0.10–7.89	0.72
Discontinuations	RR 2.40; 95% CI 1.24–4.65	RR 1.51; 95% CI 0.67–3.42	0.39

Subgroup analysis by class of immune checkpoint inhibitor	anti-CTLA-4 mAb	anti-PD-1/PD-L1 mAb	*p*-value for difference
All grade-AEs	RR 1.06; 95% CI 1.03–1.10	RR 1.00; 95% CI 0.99–1.02	0.0004
Grade 3/4 AEs	RR 1.42; 95% CI 1.14–1.79	RR 1.11; 95% CI 0.96–1.28	0.07
Deaths	RR 1.92; 95% CI 0.83–4.45	RR 1.11; 95% CI 0.67–1.86	0.28
Discontinuations	RR 3.22; 95% CI 1.66–6.23	RR 1.34; 95% CI 1.07–1.67	0.01

Based on the type of immune checkpoint inhibitor combined with CTx, the combination of CTx with an anti-CTLA-4 mAb was associated with a greater increase in all-grade AEs compared to monotherapy (RR 1.06; 95% CI 1.03–1.10). However, there were no differences when the combination with CTx was an anti-PD-1/PD-L1 mAb (RR 1.00; 95% CI 0.99–1.02). A tendency towards a higher number of grade 3/4 AEs was observed with the use of anti-CTLA4 mAb in combination compared to anti-PD-1/PD-L1 mAb, but this was not statistically significant (*p* = 0.07). Mortality was similar between the two types of ICB agents. The anti-CTLA4 combination presented more treatment discontinuations compared to anti-PD-1/PD-L1 mAb combinations with CTx (RR 3.22; 95% CI 1.66-6.23 versus RR 1.34; 95% CI 1.07–1.67, respectively).

## DISCUSSION

For many years now, oncologists have combined different drugs to achieve better outcomes. Most combination strategies have emerged empirically without considering overlapping safety profiles. Whether combination compared to sequential treatment is a better strategy overall is usually a matter for debate.

The use of ICB as monotherapy has generally shown a good safety profile without potential overlapping toxicities with CTx, and as such it seems reasonable to combine these two therapeutic strategies.

This meta-analysis has shown that the combination of ICB with CTx is associated with an increase in grade 3/4 AEs and higher rates of treatment discontinuation when compared to monotherapy in different solid tumors. Importantly, fatal adverse events (deaths) do not seem to be increased with the combination of CTx and ICB.

Subgroup analysis for the control group (CTx or ICB as monotherapy) showed no differences, confirming a worse safety profile with the use of combined treatment. This subanalysis could be limited by the small number of events in the ICB monotherapy group.

We should take into account that up to 70% of the trials in this analysis included ipilimumab. CTx plus ICB with CTLA-4 mAbs (ipilimumab) showed a significant increase in all-grade AEs and discontinuations compared to CTx and anti-PD-1/PD-L1 mAbs, probably as a consequence of the poorer safety profile observed with anti-CTLA-4 mAbs in monotherapy compared to anti-PD-1/PD-L1 [10].

Despite the worse safety profile and higher treatment discontinuation rates for the combination strategy, its efficacy has been proven. At this point, the issue is to predict which patients might really benefit from combination therapy and which might have poor tolerance. One example would be to establish if PD-L1 expression levels could select patients in some indications for monotherapy, avoiding unnecessary toxicities.

CTx plus ICB has been extensively tested in several advanced tumors, where the main goal for patients is to increase overall survival as well as to maintain quality of life. It is therefore extremely important to consider the toxicity implications of combined CTx plus ICB, and to carefully balance the risk and benefits of the new strategies.

The impact of increased treatment discontinuations on outcomes is not yet completely understood. A pooled analysis of patients with advanced melanoma who received nivolumab combined with ipilimumab concluded that efficacy outcomes were similar between patients who discontinued nivolumab plus ipilimumab treatment because of AEs during the induction phase and those who did not discontinue because of AEs. Thus, even after discontinuation, some patients may continue to benefit [25]. In addition, sequential strategies also need to be properly compared to combination therapies, to establish the actual relative benefit with these two modalities.

There are still many questions to be answered regarding the optimal dose, regimen or duration of therapy for many CTx plus ICB combinations. The development of better ways to manage toxicities also needs to be addressed, to avoid treatment discontinuations and try to maintain the proposed treatment regimen. These questions can only be explored through prospective clinical trials and extensive collaborative research.

This meta-analysis has several limitations. It includes a heterogeneous population (different tumor types or population enriched by PD-L1 expression in some studies, different combination treatments). Although rather similar, there were minor variations in the safety criteria used to evaluate and grade the adverse events (mainly CTCAE criteria versions 3.0 and 4.0). In addition, we were unable to retrieve patient-level data, although some studies have suggested trial-level and patient-level meta-analyses may reach comparable outcomes [26]. Finally, in this study, we did not evaluate the effect of maintenance strategies on the toxicity profile. In conclusion, in this meta-analysis, CTx plus ICB is associated with an increase in grade 3/4 AEs and treatment discontinuations compared to monotherapy (both CTx and ICB). Treatment-related deaths were not increased in the CTx plus ICB group. Many clinical trials are combining multiple strategies to develop the most effective combination in immuno-oncology. The identification of biomarkers that predict efficacy as well as the risks of adverse effects is essential to improve the benefit-risk balance of treatment for our patients.

## References

[1] Rittmeyer A, Barlesi F, Waterkamp D, Park K, Ciardiello F, von Pawel J, Gadgeel SM, Hida T, Kowalski DM, Dols MC, Cortinovis DL, Leach J, Polikoff J, et al; OAK Study Group Atezolizumab versus docetaxel in patients with previously treated non-small-cell lung cancer (OAK): a phase 3, open-label, multicentre randomised controlled trial. Lancet. 2017; 389:255–65. 10.1016/s0140-6736(16)32517-x. 27979383 PMC6886121

[2] Fehrenbacher L, Spira A, Ballinger M, Kowanetz M, Vansteenkiste J, Mazieres J, Park K, Smith D, Artal-Cortes A, Lewanski C, Braiteh F, Waterkamp D, He P, et al; POPLAR Study Group Atezolizumab versus docetaxel for patients with previously treated non-small-cell lung cancer (POPLAR): a multicentre, open-label, phase 2 randomised controlled trial. Lancet. 2016; 387:1837–46. 10.1016/s0140-6736(16)00587-0. 26970723

[3] Hodi FS, O’Day SJ, McDermott DF, Weber RW, Sosman JA, Haanen JB, Gonzalez R, Robert C, Schadendorf D, Hassel JC, Akerley W, van den Eertwegh AJ, Lutzky J, et al Improved survival with ipilimumab in patients with metastatic melanoma. N Engl J Med. 2010; 363:711–23. 10.1056/nejmoa1003466. 20525992 PMC3549297

[4] Ribas A, Puzanov I, Dummer R, Schadendorf D, Hamid O, Robert C, Hodi FS, Schachter J, Pavlick AC, Lewis KD, Cranmer LD, Blank CU, O’Day SJ, et al Pembrolizumab versus investigator-choice chemotherapy for ipilimumab-refractory melanoma (KEYNOTE-002): a randomised, controlled, phase 2 trial. Lancet Oncol. 2015; 16:908–18. 10.1016/S1470-2045(15)00083-2. 26115796 PMC9004487

[5] Brahmer J, Reckamp KL, Baas P, Crinò L, Eberhardt WE, Poddubskaya E, Antonia S, Pluzanski A, Vokes EE, Holgado E, Waterhouse D, Ready N, Gainor J, et al Nivolumab versus Docetaxel in Advanced Squamous-Cell Non-Small-Cell Lung Cancer. N Engl J Med. 2015; 373:123–35. 10.1056/nejmoa1504627. 26028407 PMC4681400

[6] Motzer RJ, Escudier B, McDermott DF, George S, Hammers HJ, Srinivas S, Tykodi SS, Sosman JA, Procopio G, Plimack ER, Castellano D, Choueiri TK, Gurney H, et al; CheckMate 025 Investigators Nivolumab versus Everolimus in Advanced Renal-Cell Carcinoma. N Engl J Med. 2015; 373:1803–13. 10.1056/NEJMoa1510665. 26406148 PMC5719487

[7] Sharma P, Hu-Lieskovan S, Wargo JA, Ribas A Primary, Adaptive, and Acquired Resistance to Cancer Immunotherapy. Cell. 2017; 168:707–23. 10.1016/j.cell.2017.01.017. 28187290 PMC5391692

[8] Larkin J, Chiarion-Sileni V, Gonzalez R, Grob JJ, Cowey CL, Lao CD, Schadendorf D, Dummer R, Smylie M, Rutkowski P, Ferrucci PF, Hill A, Wagstaff J, et al Combined Nivolumab and Ipilimumab or Monotherapy in Untreated Melanoma. N Engl J Med. 2015; 373:23–34. 10.1056/nejmoa1504030. 26027431 PMC5698905

[9] Wolchok JD, Chiarion-Sileni V, Gonzalez R, Rutkowski P, Grob JJ, Cowey CL, Lao CD, Wagstaff J, Schadendorf D, Ferrucci PF, Smylie M, Dummer R, Hill A, et al Overall Survival with Combined Nivolumab and Ipilimumab in Advanced Melanoma. N Engl J Med. 2017; 377:1345–56. 10.1056/nejmoa1709684. 28889792 PMC5706778

[10] De Velasco G, Je Y, Bossé D, Awad MM, Ott PA, Moreira RB, Schutz F, Bellmunt J, Sonpavde GP, Hodi FS, Choueiri TK Comprehensive Meta-analysis of Key Immune-Related Adverse Events from CTLA-4 and PD-1/PD-L1 Inhibitors in Cancer Patients. Cancer Immunol Res. 2017; 5:312–8. 10.1158/2326-6066.cir-16-0237. 28246107 PMC5418853

[11] Schwarzer G Meta: An R package for meta-analysis. R News. 2007; 7:40–5.

[12] R Core Team R: A language and environment for statistical computing. R Foundation for Statistical Computing Vienna, Austria 2016 Available from: https://www.R-project.org/.

[13] Higgins JP, Thompson SG, Deeks JJ, Altman DG Measuring inconsistency in meta-analyses. BMJ. 2003; 327:557–60. 10.1136/bmj.327.7414.557. 12958120 PMC192859

[14] DerSimonian R, Laird N Meta-analysis in clinical trials. Control Clin Trials. 1986; 7:177–88. 10.1016/0197-2456(86)90046-2. 3802833

[15] Reck M, Luft A, Szczesna A, Havel L, Kim SW, Akerley W, Pietanza MC, Wu YL, Zielinski C, Thomas M, Felip E, Gold K, Horn L, et al Phase III Randomized Trial of Ipilimumab Plus Etoposide and Platinum Versus Placebo Plus Etoposide and Platinum in Extensive-Stage Small-Cell Lung Cancer. J Clin Oncol. 2016; 34:3740–8. 10.1200/jco.2016.67.6601. 27458307

[16] Reck M, Bondarenko I, Luft A, Serwatowski P, Barlesi F, Chacko R, Sebastian M, Lu H, Cuillerot JM, Lynch TJ Ipilimumab in combination with paclitaxel and carboplatin as first-line therapy in extensive-disease-small-cell lung cancer: results from a randomized, double-blind, multicenter phase 2 trial. Ann Oncol. 2013; 24:75–83. 10.1093/annonc/mds213. 22858559

[17] Lynch TJ, Bondarenko I, Luft A, Serwatowski P, Barlesi F, Chacko R, Sebastian M, Neal J, Lu H, Cuillerot JM, Reck M Ipilimumab in combination with paclitaxel and carboplatin as first-line treatment in stage IIIB/IV non-small-cell lung cancer: results from a randomized, double-blind, multicenter phase II study. J Clin Oncol. 2012; 30:2046–54. 10.1200/jco.2011.38.4032. 22547592

[18] Govindan R, Szczesna A, Ahn MJ, Schneider CP, Gonzalez Mella PF, Barlesi F, Han B, Ganea DE, Von Pawel J, Vladimirov V, Fadeeva N, Lee KH, Kurata T Phase III Trial of Ipilimumab Combined With Paclitaxel and Carboplatin in Advanced Squamous Non-Small-Cell Lung Cancer. J Clin Oncol. 2017; 35:3449–57. 10.1200/jco.2016.71.7629. 28854067

[19] Langer CJ, Gadgeel SM, Borghaei H, Papadimitrakopoulou VA, Patnaik A, Powell SF, Gentzler RD, Martins RG, Stevenson JP, Jalal SI, Panwalkar A, Yang JC, Gubens M, et al; KEYNOTE-021 investigators Carboplatin and pemetrexed with or without pembrolizumab for advanced, non-squamous non-small-cell lung cancer: a randomised, phase 2 cohort of the open-label KEYNOTE-021 study. Lancet Oncol. 2016; 17:1497–508. 10.1016/s1470-2045(16)30498-3. 27745820 PMC6886237

[20] Hersh EM, O’Day SJ, Powderly J, Khan KD, Pavlick AC, Cranmer LD, Samlowski WE, Nichol GM, Yellin MJ, Weber JS A phase II multicenter study of ipilimumab with or without dacarbazine in chemotherapy-naïve patients with advanced melanoma. Invest New Drugs. 2011; 29:489–98. 10.1007/s10637-009-9376-8. 20082117

[21] Robert C, Thomas L, Bondarenko I, O’Day S, Weber J, Garbe C, Lebbe C, Baurain JF, Testori A, Grob JJ, Davidson N, Richards J, Maio M, et al Ipilimumab plus dacarbazine for previously untreated metastatic melanoma. N Engl J Med. 2011; 364:2517–26. 10.1056/nejmoa1104621. 21639810

[22] Weber J, Hamid O, Amin A, O’Day S, Masson E, Goldberg SM, Williams D, Parker SM, Chasalow SD, Alaparthy S, Wolchok JD Randomized phase I pharmacokinetic study of ipilimumab with or without one of two different chemotherapy regimens in patients with untreated advanced melanoma. Cancer Immun. 2013; 13:7. 23833564 PMC3700777

[23] Gandhi L, Rodríguez-Abreu D, Gadgeel S, Esteban E, Felip E, De Angelis F, Domine M, Clingan P, Hochmair MJ, Powell SF, Cheng SY, Bischoff HG, Peled N, et al Pembrolizumab plus Chemotherapy in Metastatic Non-Small-Cell Lung Cancer. N Engl J Med. 2018; 378:2078–92. 10.1056/nejmoa1801005. 29658856

[24] Socinski MA, Jotte RM, Cappuzzo F, Orlandi F, Stroyakovskiy D, Nogami N, Rodríguez-Abreu D, Moro-Sibilot D, Thomas CA, Barlesi F, Finley G, Kelsch C, Lee A, et al; IMpower150 Study Group Atezolizumab for First-Line Treatment of Metastatic Nonsquamous NSCLC. N Engl J Med. 2018; 378:2288–301. 10.1056/nejmoa1716948. 29863955

[25] Schadendorf D, Wolchok JD, Hodi FS, Chiarion-Sileni V, Gonzalez R, Rutkowski P, Grob JJ, Cowey CL, Lao CD, Chesney J, Robert C, Grossmann K, McDermott D, et al Efficacy and Safety Outcomes in Patients With Advanced Melanoma Who Discontinued Treatment With Nivolumab and Ipilimumab Because of Adverse Events: A Pooled Analysis of Randomized Phase II and III Trials. J Clin Oncol. 2017; 35:3807–14. 10.1200/jco.2017.73.2289. 28841387 PMC5791828

[26] Bennett CL Venous thromboembolism and mortality associated with recombinanterythropoietin and darbepoetin administration for the treatment of cancer-associated anemia. JAMA. 2008; 299:914. 10.1001/jama.299.8.914. 18314434

